# Adjuvant Effects of *L. acidophilus* LW1 on Immune Responses to the Foot-and-Mouth Disease Virus DNA Vaccine in Mice

**DOI:** 10.1371/journal.pone.0104446

**Published:** 2014-08-13

**Authors:** Junhong Su, Jidong Li, Haixue Zheng, Yanan You, Xuenong Luo, Yungang Li, Xueqiang Li, Xusheng Ma, Junjun Li, Yongxi Dou, Xuepeng Cai

**Affiliations:** 1 State Key Laboratory of Veterinary Etiological Biology, Lanzhou Veterinary Research Institute, Chinese Academy of Agricultural Sciences (CAAS), Lanzhou, PR China; 2 School of Agriculture, Ningxia University, Yinchuan, RP China; 3 Shandong Provincial Center for Animal Disease Prevention and Control, Jinan, PR China; Nanyang Technological University, Singapore

## Abstract

The adjuvant effects of *Lactobacillus acidophilus* on DNA vaccination are not fully understood. It has been hypothesized that swine-derived *Lactobacillus acidophilus* SW1 (LASW1) could function as an immune adjuvant to enhance antigen-specific immune responses after foot-and-mouth disease (FMD) DNA vaccination in mice. To evaluate the effect of oral LASW1 on the immune response to a DNA vaccine (pRC/CMV-vp1) harboring FMD VP1 gene, anti-FMDV antibody and its isotypes, T-cell proliferation, and cytokine detection were investigated. The results showed that LASW1 was able to enhance FMDV-specific antibody levels and FMDV-neutralizing antibodies. After a booster vaccine, the anti-FMDV antibody titers and FMDV-neutralizing antibodies levels induced by pRC/CMV-vp1 were higher in mice treated with LSAW1 than in the group immunized with pRC/CMV-vp1 alone (the control). Using T-cell proliferation, the stimulation index of the LASW1 group was significantly higher in response to ConA and 146S antigen (*P*<0.05) than in the control group. Importantly, higher concentrations of IFN-γ and IFN-γ-producing cells were also observed in splenocytes isolated from the experimental LASW1 mice, indicating that INF-γ secretion is important to the immune response to LASW1. The results indicate that LASW1 is a promising immune adjuvant in DNA vaccination against FMD when administrated orally.

## Introduction

Foot-and-mouth disease (FMD) is one of the most contagious and economically devastating viral diseases of cloven-hoofed livestock worldwide. The causative agent of FMD is the foot-and-mouth disease virus (FMDV). It has a single-stranded, plus-sense RNA genome of approximately 8500 bases surrounded by four structural proteins. This virus is in the Aphthovirus genus of the Picornaviridae family [1]. To date, a chemically inactivated whole FMDV vaccine formulated with oil adjuvant is the most widely used means of FMD control in China and other countries. However, conventional FMD vaccine production requires advanced containment facilities and involves some danger that live virus may escape [2]. DNA vaccines are one alternative to conventional vaccines. These vaccines deliver gene-encoding protein antigens to host cells, and antigen production occurs *in vivo* [3]. DNA vaccines have elicited protective immune responses against a variety of pathogenic agents, including HIV, influenza, FMDV, and tumor cells [4]–[7]. Despite this progress, DNA vaccines often generate weak immune responses, resulting in either partial protection or failure [8]–[10]. Effective adjuvants and delivery systems are needed to improve immune responses elicited by DNA vaccines. Cytokines and co-stimulatory molecules are potential adjuvants and may improve the efficacy of DNA vaccines [11], [12].

Probiotics, such as lactic acid bacteria (LAB), are generally regarded as safe (GRAS) and confer a health benefit on the host [13]. LAB have been used to prevent and treat gastrointestinal disorders in both humans and other animals [14]. Recent studies have demonstrated that *Lactobacillus acidophilus* is a strong Th1 cytokine (IL-12, IFN-γ) inducer [15], [16]. *L. acidophilus* strongly up-regulates surface markers on dendritic cells (DCs), including CD40, CD86, CD83, and HLA-DR [16]. *Lactobacillus* has been shown to increase antigen-specific immune responses induced by viral or bacterial vaccines, including influenza [17] and diphtheria [18]. One previous study demonstrated that the swine-derived *L. acidophilus* SW1 (LASW1) strain can act as an immune adjuvant when used as a living carrier for a DNA vaccine against FMD [19]. The observed response may have been mediated in part by *L. acidophilus*-mediated alterations in systemic cytokine secretion, although this was not formally tested.

In the current study, LASW1 was used as an oral adjuvant to the FMDV VP1 DNA vaccine (pRC/CMV-vp1). The ability of LASW1 to enhance both FMDV-specific humoral and cellular responses was evaluated. The effects of LASW1 on IFN-γ production in splenotytes isolated from the immunized mice, which may correlate the innate and adaptive activations, were also assessed. Results showed both humoral and cellular responses to have been significantly enhanced in pRC/CMV-vp1-immunized mice given oral LASW1. In this way, swine-derived LASW1 can enhance the immune response to FMD DNA vaccines.

## Materials and Methods

### 2.1 Construction of pRc/CMV-VP1 plasmid

To construct a DNA VP1 vaccine, the sequence of VP1 was amplified by PCR using PRC product P12A-3C (type O strain, O/BY/CHA/2010, GenBank Accession no: JN998085.1) [20]. The PCR product VP1 was inserted into pMD18-Teasy vector (TaKaRa) for sequencing analysis. The genes were then sub-cloned into a pRC/CMV2 vector (Invitrogen) and designated as pRC/CMV-VP1.

### 2.2 VP1 gene expression in BHK cells

Baby hamster kidney cells (BHK-21) were cultured in Dulbecco's modified Eagle's medium (DMEM) supplemented with 10% fetal calf serum (FCS) in a six-well tissue culture plate under 5% CO_2_ at 37°C. BHK-21 cells were transfected using the X-tremeGENE HP DNA transfection reagent (Roche) per the manufacturer's instructions. In brief, 2 μg plasmid pRc/CMV-VP1 in 100 μl OPTI (Invitrogen) was mixed with 6 μl X-tremeGENE HP DNA transfection reagent at room temperature and incubated for 20 min. The DNA/transfection reagent complexes were slowly added to cells and incubated for 48 h. The cells in each well were fixed with an ice-cold acetone and methanol mixture (1∶1) for 30 min at -20°C. After being washed three times with 0.01 M pH 7.2 Phosphate Buffered Saline with Tween 20 (PBST) (containing 0.05% Tween-20), primary rabbit anti-FMDV type O-IgG antibody (LVRI) (1∶50 Phosphate Buffered Saline, PBS) was applied to the wells for 1 h at 37°C. The cells were then washed three times and incubated with FITC-conjugated goat anti-rabbit-IgG (Sigma) (1∶200 in PBS) for 30 min at 37°C. The cells were then washed five more times. The cells were examined under an Olympus fluorescence microscope.

### 2.3 Bacteria

*L. acidophilus* swine strain (LASW1) was derived from the intestine of pathogen-free swine [19]. The bacteria were cultured on a MRS agar (BD Bioscience, San Jose, CA, USA) plate at 37°C, 5% CO_2_ for 24–48 h. The bacterial colony was then transferred to MRS broth medium (BD Bioscience) and maintained under the same conditions for 16–24 h. The cells were harvested at 6000 rpm for 10 min when the OD600 ≈0.6, washed three times with fresh phosphate-buffered saline (PBS), and then re-suspended with PBS.

### 2.4 Animal and immunization

BALB/c mice six to eight weeks of age were obtained from the Animal Center for Lanzhou Veterinary Research Institute (LVRI). Before the beginning of the experiment, the mice were acclimatized for one week. All animals were handled in strict accordance with good animal practice as stipulated by the Animal Ethics Procedures and Guidelines of the People's Republic of China, and the study was approved by the Animal Ethics Committee of LVRI, Chinese Academy of Agricultural Sciences (Permit No. LVRIAEC2010-006). For experimental animal grouping, 24 male mice were randomly divided into five groups of 6 mice each. In the experimental group, each mouse received an oral dose of bacteria (2–5×10^9^ CFU) 5 days after intramuscular administration of 50 μg plasmid pRC/CMV-VP1, which itself took place 20 min after an injection of 25% glucose 50 μl at the same site. Mice received the same amount of pRC/CMV-VP1 or vector pRC/CMV or an oral dose of bacteria (2–5×10^9^ CFU) 5 days or commercial inactivated foot-and-mouth disease virus (type O) vaccine (50 μl each mouse, supplied by China Animal Husbandry Industry Co., Lanzhou, China) were treated as controls. A booster immunization with 50 μg pRC/CMV-VP1 alone to the experimental group was given 21 days after the initial immunization. To the controls, vaccination of the same amount of pRC/CMV-VP1 or vector pRC/CMV or FMD vaccine served as the booster immunization. Mouse sera were collected from tail vein two weeks after the last immunization and stored at −80°C until use.

### 2.5 FMDV-specific IgG and IgG isotypes

Serum samples were analyzed for IgG and levels of isotypes using an indirect double antibody sandwich enzyme-linked immunosorbent assay (DAS-ELISA) as described previously [21]. In brief, the wells of polyvinyl 96-well microliter plates were filled with 50 μl rabbit anti-FMDV (type O or Asia1) antibody (LVRI) in 0.05 M carbonate/bicarbonate buffer pH 9.6 (1∶1000) and incubated overnight at 4°C. After washing with PBS containing 0.05% Tween-20 (PBST), the wells were incubated with 3% skimmed milk for blockage at 37°C for 2 h. Then the wells were filled with 50 μl FMDV antigen (LVRI) (1∶4 dilution) and incubated at 4°C for 2 h. The plates were washed five times. Then the wells were filled with 50 μl serum (diluted serially for IgG or diluted 1∶10 for isotype analysis in PBS 5% skim milk) and incubated at 37°C for 1 h. Plates were then washed five times with PBST. For IgG level detection, 50 μl of goat anti-mouse IgG (1∶1000) (Sigma) was added to the wells and incubated at 37°C for 1 h. Fifty microliters of 3,3,5,5-tetramethyl benzidine solution (Sigma) was added to each well after washing, and plates were incubated in the dark at 37°C for approximately 15 min. The reaction was stopped by adding 50 μl 2 M H_2_SO_4_. The absorbance of each well at 450 nm was read using a microplate reader (BioRad). Values above the cut-off background level (mean value of sera from pRC/CMV2-immunized mice multiplied by a factor of 2.1) were considered positive. The antibody levels were expressed as the optical density values (OD450) of the mouse sample. Titers were depicted as reciprocal end-dilutions. For subclasses, 50 μl of goat anti-mouse IgG1 or IgG2a (1∶1000) (Sigma) was used to the corresponding plates and then incubated for 1 h at 37°C.

### 2.6 FMDV-neutralizing antibodies

Virus neutralization test (VNT) ware carried out in 96 wells as described in the OIE Manual of Diagnostic Tests and Vaccines for Terrestrial Animals [22]. Serial dilutions of sera were performed in duplicate and 50 μl of each were added for 1 h to 50 μl 100 TCID_50_ of FMDV, type O strain, O/BY/CHA/2010. BHK-21 cell suspension was then added to each well and the plates were incubated at 37°C for 3 days. The cells were fixed with formalin and stained with methylene blue. Titers were expressed as the last serum dilution that inhibited viral replication in 50% of the wells.

### 2.7 Splenocyte proliferation assay

Two weeks after booster immunization, three mice from each group were killed by cervical dislocation. Splenic lymphocytes were isolated from test and control groups using Mouse 1× Separation Medium (Dakewe Biotech Company, Shenzhen, China). A single-cell suspension was then prepared and re-suspended at 3–5×10^5^ cells/ml in RPMI 1640 containing 10% fetal calf serum. The splenocytes were plated out in triplicate, with 100 μl of the dilutions placed into 96-well microplate and cultured for 24 h. The cells were then stimulated for 48 h with 5 μg/ml of ConA, 2 μg/ml of 146S antigen, 2 μg/ml BSA (irrelevant control) and medium (negative control), respectively. Cell proliferation was verified using the CellTiter 96 AQueous One Solution Cell Proliferation assay (Promega), as previously reported [23]. Briefly, 20 μl of the CellTiter 96 AQueous One Solution reagent was added to each well, and the plate was incubated at 37°C in 5% CO_2_ for 1–4 h. Absorbance values for each well were measured at 490 nm using a microplate reader (BioRad). Data are expressed as the stimulation index (SI), calculated as the mean OD value of wells stimulated with an antigen, divided by the mean OD value of wells treated with medium.

### 2.8 *In vitro* cytokine detection

For analysis of cytokine IFN-γ and il-4 *in vitro* production, single splenocytes were isolated and prepared as above from the test and control groups. Cells from each group were cultured with 146S antigen (2 μg/ml) in triplicate wells under 5% CO_2_ at 37°C for 48 h. The cytokine concentration in the supernatant of the splenocytes was measured using commercial ELISA kits (eBioscience) according to the manufacturer's instructions in duplicate against a standard curve.

### 2.9 ELISpot assay

Mouse IFN-γ ELISpot pre-coated kits were used (Dakewe Biotech Company, Shenzhen, China) according to manufacturer's instructions. Briefly, 200 μl of Lympho-SpotTM medium was added to all wells to activate the plate for 10 min at room temperature (RT), and then the medium was discarded. Splenocytes were prepared (as above) as above from the immunized male mice (n = 3) with or without (control) oral administration of LASW1 at a density of 1×10^5^ cells/well in 10% RPME medium, and they were stimulated with 5 μg/ml phytohemagglutinin (PHA) or 2 μg/ml of 146S antigen, 5 μg/ml BSA (negative control), or Lympho-spot™ medium (blank control). These were incubated with 5% CO2 at 37°C for 16–36 h. After discarding the cells and medium, 200 μl of DI water was added to each well and incubated for 10 min at 4°C for cell lysis. The wells were then washed 5–7 times with washing buffer. One hundred microliters of bio-conjugated antibody was added to all wells and incubated for 1 h at 37°C. After washing, 100 μl of streptavidin-HPR was added to all wells and incubated for 1 h at 37°C. After another washing, 100 μl of ingrain agent was added to the wells and incubated for 15–45 min at RT. In order to stop the reaction, the plates were washed with DI water twice and then dried for 10–30 min at RT. The number of spots per well was determined using an automatic plate reader, Biosys Bioreader 4000 (Biosys, Germany).

### 3.0 Statistical analysis

Statistical analysis was performed using SPSS Statistics 19.0 software, and a one-way ANOVA was used to calculate significance. *P* < 0.05 was considered statistically significant and is indicated with one asterisk in figures.

## Results

### 3.1 Expression of VP1 gene in BHK cells

**Figure 1:**
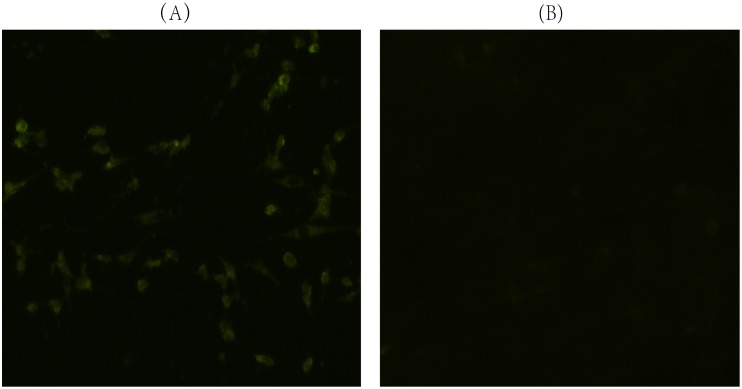
Expression of VP1 protein in BHK cells. Primary rabbit anti-FMDV type O-IgG antibody (LVRI) was applied to the cells, which were then incubated with FITC-conjugated goat anti-rabbit-IgG (Sigma). (A) BHK cells transfected with the eukaryotic plasmid pRC/CMV-VP1 show typical cytoplasm immunospecific fluorescence. (B) BHK cells transfected with pRc/CMV alone express no immunofluorescence.

To confirm the expression of pRC/CMV-VP1 in a eukaryotic system, the plasmids were transfected into BHK cells. As shown in Fig. 1, in the pRc/CMV-VP1 transfected BHK cell line, immunospecific fluorescence was brightest in cytoplasm regions. In the control, no fluorescence signal was emitted in the BHK cells. The results demonstrated that pRc/CMV-VP1 can express FMDV VP1 protein in BHK cells.

### 3.2 LASW1 improves humoral immune responses

**Figure 2:**
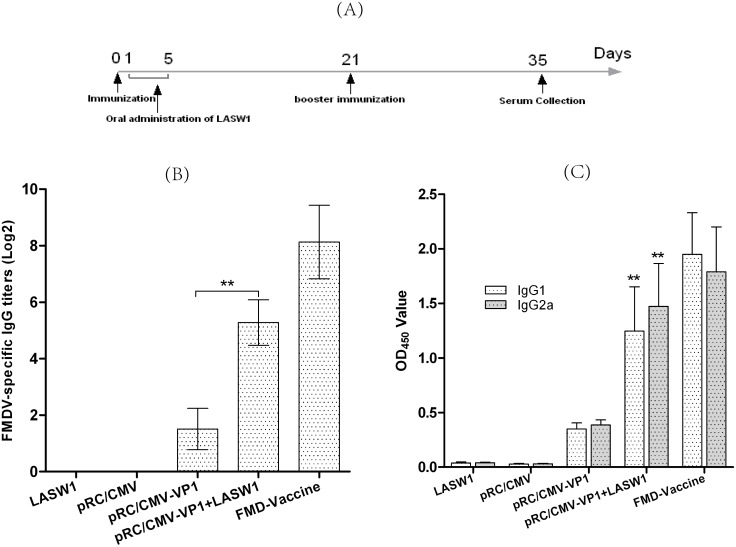
FMDV-specific humoral response. (A) Graphic timeline in the study from animal immunization to sera collection. (B) FMDV-specific IgG titers. Asterisks (*) indicate the values of pRC/CMV-VP1- and LASW1-treated group were statistically different than mice immunized with pRC/CMV-VP1 alone (*P*<0.05). (C) Effects of LASW1 on the level of FMDV-specific IgG isotypes. The levels of IgG isotypes were expressed as the optical density values at 450 nm (OD450) of mice sample. Asterisks (**) indicate that the values of pRC/CMV-VP1- and LASW1-treated group were statistically different from those of mice immunized with pRC/CMV-VP1 alone (*P*<0.01).

To examine the effects of LASW1 on the humoral responses against FMD, Babl/c mice were given LASW1 orally after immunization with pRC/CMV-VP1. Serum samples were collected 14 days after the second immunization, and the level of total IgG, IgG1, and IgG2a antibodies were detected using DAS-ELISA. Fig. 2A indicates that FMDV-specific IgG titers were higher in mice treated with pRC/CMV-VP1 plus LASW1 than in mice treated with pRC/CMV-VP1 alone (*P*<0.01). The treatment group showed significantly higher levels of IgG1 and IgG2a than the group immunized with pRC/CMV-VP1 alone (Fig. 2B). These data indicate that oral LASW1 can improve the antigen-specific humoral response in mice immunized with pRC/CMV-VP1.

### 3.3 Virus neutralization test

**Figure 3:**
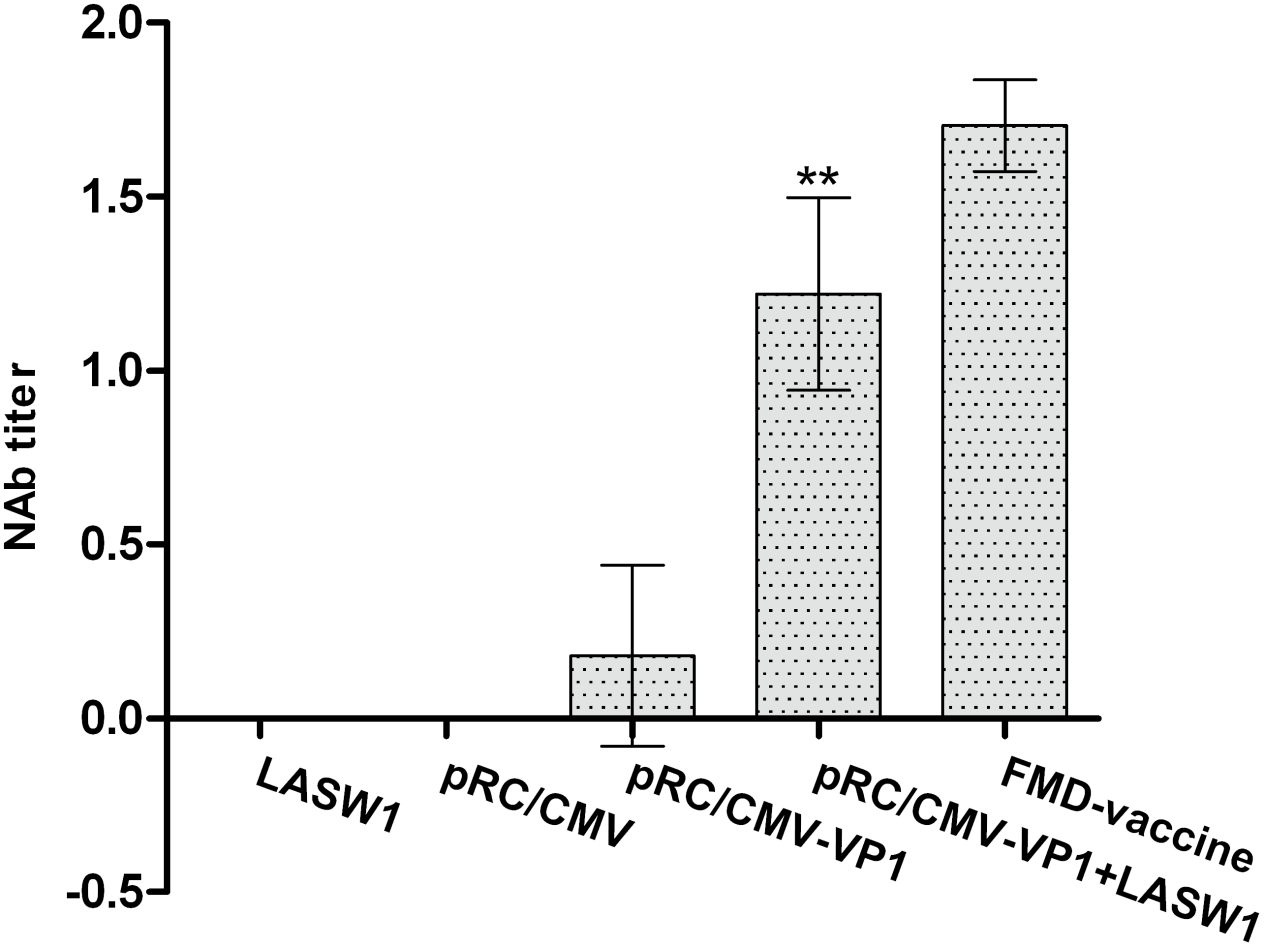
Induction of anti-FMDV-neutralizing antibodies. Mean titers ±SD are shown. Asterisks (*) indicate that the values of pRC/CMV-VP1- and LASW1-treated group were statistically different from those of mice immunized with pRC/CMV-VP1 alone (*P*<0.05).

A VNT assay was performed in order to confirm the levels of FMDV-neutralizing antibodies in the pRC/CMV-VP1- and LASW1-treated groups. As a result, higher levels of neutralizing antibodies were observed in the group injected with pRC/CMV-VP1 and given oral LASW1 than in the groups given either pRC/CMV-VP1 or LASW1 alone (Fig. 3). However, there were lower levels of FMDV-neutralizing antibodies in the group given both pRC/CMV-VP1 and LASW1than in the FMD vaccine group.

### 3.4 T-cell proliferative response

**Figure 4:**
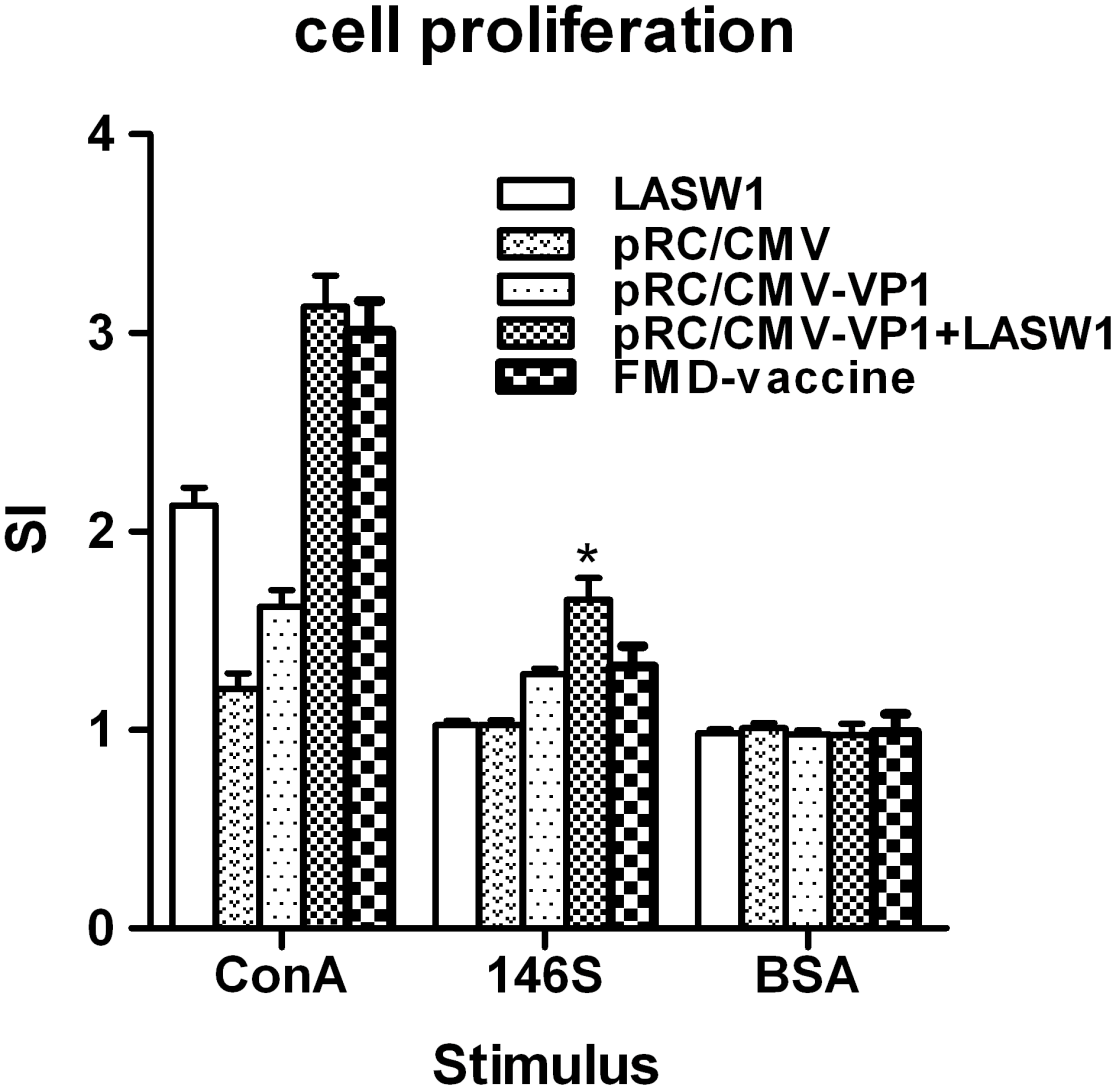
Effects of oral LASW1 on T-cell proliferation. Single splenocyte suspension was isolated from animals 14 days after the booster immunization, plated in triplicate in a 96-well plate and stimulated in vitro for 48 h with 146S antigen, ConA (positive control), BSA (irrelevant control), and medium (negative control). Proliferation was analyzed by the MTS assay and expressed as stimulation index.

An MTS assay was used to assess T-cell proliferation. To further determine whether oral LASW1 influences cell-mediated immunity. As shown in Fig. 4, oral LASW1 resulted in significantly more T cells than pRC/CMV-VP1, pRC/CMV, or LASW1 alone. The splenocyte proliferation response to ConA was stronger in the mice treated with LASW1 than in control mice. These results suggest that oral LASW1 can enhance the antigen-specific T-cell response.

### 3.5 Cytokine expression in T cells

**Figure 5:**
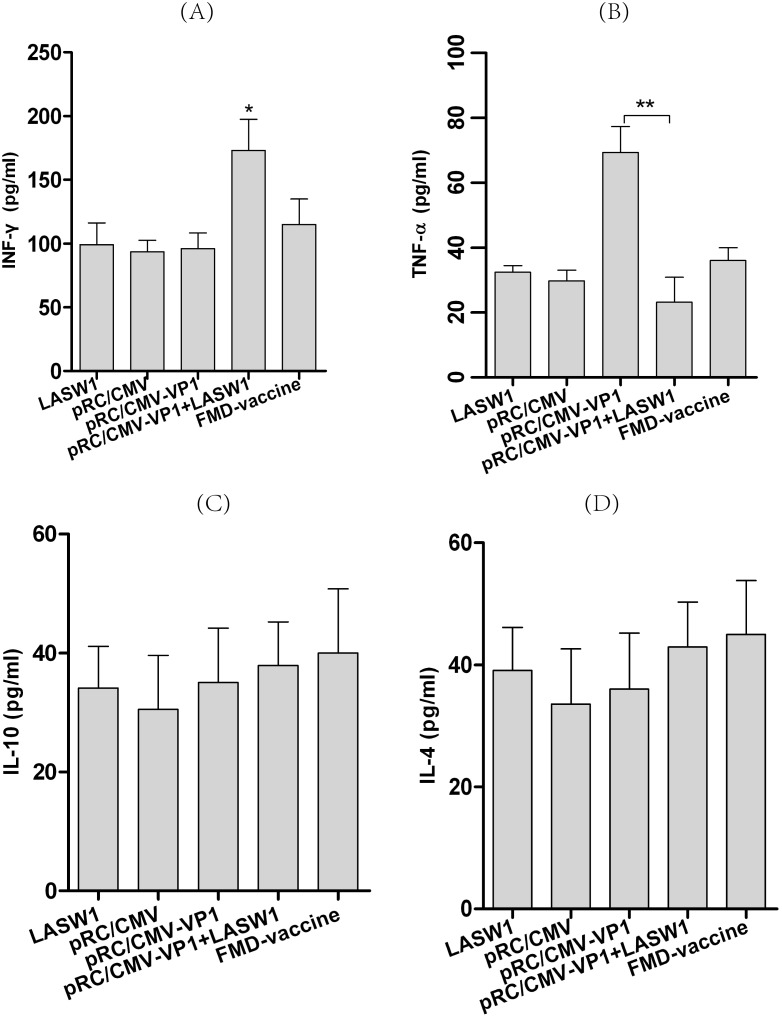
IFN-γ, TNF-α, IL-4, and IL-10 levels as measured by ELISA. Splenocytes prepared from LASW1 treated or control mice immunized with pRC/CMV-VP1 were cultured with 146S antigen (2 μg/ml). The concentrations of (A) IFN-γ, (B) TNF-α, (C) IL-4 and (D) IL-10 in culture supernatants were measured at 48 h. Bars represent the mean ± SD of triplicate wells. *P<0.05, **P<0.01.

To detect the production of cytokines *in vitro*, 14 days after booster vaccination, splenocytes from three mice from each group were cultured with 146S antigen or medium as a negative control. As shown in Fig. 5A, higher concentrations of IFN-γ were observed in pRC/CMV-VP1-immunized mice treated with oral LASW1 than in the control mice. This suggested that oral administration of LASW1 enhanced secretion of IFN-γ from splenocytes. There was no difference in IL-4 or IL-10 production between the two groups.

### 3.6 INF-γ ELISpot assay

**Figure 6:**
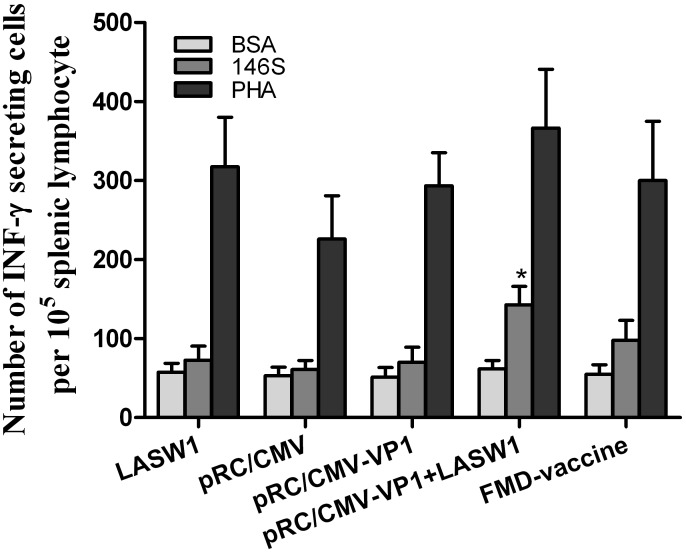
Concentration of IFN-γ-secreting cells in the mice immunized with pRC/CMV-VP1 with or without oral administration of LASW1. ELISpot assay was used to determine the number of cells secreting IFN-γ in the spleen. A single-cell suspension of lymphocytes was prepared and stimulated in culture with 146S antigen, PHA as positive control, and BSA as an irrelevant antigen. Results represent mean ± SD of number of IFN-γ producing cells of 3 male mice per group in triplicate wells.

To further confirm *in vitro* INF-γ production, an ELISpot assay determined the number of INF-γ-producing cells. As shown in Fig. 6, the number of INF-γ-producing splenic lymphocytes was significantly higher in mice treated with pRC/CMV-VP1 plus LASW1 than in mice treated with pRC/CMV-VP1 alone. These results indicate that oral LASW1 increased the number of antigen-specific INF-γ-producing cells in the spleen.

## Discussion

In this study, LASW1 as an oral adjuvant to DNA vaccination against FMDV was investigated in Babl/c mice. Plasmid pRC/CMV-VP1 was able to successfully express FMD VP1 protein in BHK cells. It was determined that LASW1 can be used as an adjuvant for enhancing both humoral and cellular responses when administered orally. Immunization with pRC/CMV-VP1 plus oral LASW1 induced a higher level of IgG production, FMDV-neutralizing antibodies, and T-cell proliferation than pRC/CMV-VP1 alone. Furthermore, the production of IFN-γ in splenocytes isolated from LASW- and pRC/CMV-VP1-treated mice was observed when stimulated with 146S.

The efficacy of vaccination against FMD is generally evaluated by humoral immune responses [24], [25]. As shown in Fig. 2 and 3, oral administration of LASW1 not only significantly increased serum FMDV-specific IgG levels but also increased the level of FMDV-neutralizing antibodies in the group injected with pRC/CMV-VP1. These results indicated that LASW1 was able to improve humoral immunity when used as an adjuvant in FMD DNA vaccine. However, cellular immune responses play an important role in the host response to intracelluar pathogens. Increasing amounts of evidence indicated that T-cell-mediated immunity is required for protection against FMD in animals [26]. Fig. 4 shows that LASW1 significantly enhanced ConA- and FMDV-stimulated splenocyte proliferation in the immunized mice, suggesting that both T and B cells had been activated.

*Lactobacillus* has been widely utilized to modulate or increase the immune response against infectious agents. Oral *Lactobacillus* can increase poliovirus neutralizing antibody titers and affect the formation of poliovirus-specific IgA and IgG in serum [27]. Virus-specific IFN-γ-producing CD8+ T-cell responses in the spleen and IgG antibody titers produced by an attenuated human rotavirus oral vaccine with *L. acidophilus* (LA) colonization are significantly higher in neonatal gnotobiotic pigs than in immunized pigs without LA colonization [28]. Li et al. clearly demonstrated that *L. acidophilus* SW1 (LASW1) as a DNA vaccine carrier can enhance both humoral and cellular immunity against FMD in Balb/c mice immunized intramuscularly [19]. Taken together, these results suggest that *Lactobacillus* can be used orally for immunomodulation and as an adjuvant to increase humoral and cellular immunity against viral infection in human and animals. *Lactobacillus* can elicit innate and adaptive immune responses in the host via binding to pattern recognition receptors, which recognize conserved molecular structures known as microbe-associated molecular patterns. This then signals (e.g., via Toll-like receptors and nucleotide oligomerization domain-like receptors) to induce the production of cytokines (such as IFN-γ and IL-12) and other innate effectors [29]. In this study, as shown in Fig. 5A, IFN-γ induced by oral administration of LASW1 was found to play a critical role in enhancing immune responses against FMD in pRC/CMV-VP1-vaccinated mice. Because it is an immunoregulatory cytokine, IFN-γ influences both the growth and differentiation of immunologically active cells [30]. Its effects include but are not restricted to enhancing CD4+ T-helper type (Th) 1 cell growth, inhibiting CD4+ Th2 cell growth [31], and stimulating murine B-cell IgG2a production [32]. In this regard, from Fig. 4, Fig. 5A, and Fig. 6, we may deduce that a higher FMDV-specific cellular immune response was likely caused by the changed production of cytokines (such as INF-γ) as a consequence of oral administration of LASW1. The effects of capacity to enhance antigen-specific immune responses likely resulted from the cytokine-inducing properties of *Lactobacillus* [33]. A higher level of TNF-α was observed in the group injected with pRC/CMV-VP1, suggesting that inflammation may be caused by injection of DNA vaccine in mice. However, the increased level of TNF-α was suppressed via oral administration of LSAW1 (Fig. 5B). These results are consistent with those of a previous study that showed LAB to have an anti-inflammatory effect on the host [34]. However, the precise mechanisms by which LASW1 acts as an adjuvant or by which it immunomodulates DNA vaccination are still unknown. This merits further investigation.

Unlike in previous studies, LASW1 was administered orally rather than as a DNA vaccine carrier for intramuscular vaccination, avoiding the instability of replication of recombinant DNA plasmid in LASW1 [19]. From a practical point of view, this may be more effective because the vaccinating plasmid has not to be adapted into the bacterial environment and it may be possible to add this adjuvant directly to DNA vaccine candidates without modifying the plasmid backbones. This new strategy may make it possible to develop an adjuvant diet that can be used to enhance the efficacy of DNA vaccines.

In conclusion, the data presented in this study clearly demonstrate that oral administration of LASW1 can enhance the immune response to DNA vaccination, and LASW1 is an effective adjuvant for the FMDV vaccine when orally administered. However, this strategy should be evaluated independently in cattle and swine.
